# Supplementary material for: Female sexual function index for screening of female sexual dysfunction using DSM-5-TR criteria in Thai women: A prospective cross-sectional diagnostic study
Source: PLoS One. 2024 Feb 22;19(2):e0298935. doi: 10.1371/journal.pone.0298935 (PMC10883536; doi:10.1371/journal.pone.0298935)
Supplement: Attachment — Submitted filename: PONE-D-23-39011 review.pdf [file pone.0298935.s002.pdf]

Thank you for the privilege of reviewing this manuscript. This manuscript reports on a validation study of the Thai version of the FSFI to assess female sexual function. Overall, this paper is well-written; a few minor edits are suggested below, given the lack of a priori criteria.

Table 4

1. Cutoff scores of 23.1, 24.1, and 23.5 are listed, but not for the same groups of participants. Please include all observed cutoff scores for: all participants, age ≤40 years, age > 40 years, premenopause, and postmenopause. It is difficult to see the benefits and limitations of the listed cutoff scores because information is omitted (e.g., women >40 years with a cutoff of 23.1, etc.).

Discussion

A cutoff score of 23.1 for screening of FSD using the DSM-5-TR criteria was considered best; however, this resulted in a sensitivity of 75.6 and specificity of 67.7, which seem a bit low. Nearly a quarter of cases are being missed with a sensitivity of only 75.6, and a third of identified cases are incorrectly identified (specificity of 67.7).

2. Please suggest secondary screeners or alternative approaches to utilizing this screener, given the low sensitivity and specificity values.
3. Please include as a limitation the lack of predetermined criteria to identify appropriate cutoff scores.
4. Please reference other measures with similar psychometric properties given the utilized cutoff score; if other measures do not use a cutoff score with 76% sensitivity and 68% specificity, please justify why a cutoff score of 23.1 is suitable as a screener for this particular measure.
